# Separation and Analysis of Aspirin and Metformin HCl Using Green Subcritical Water Chromatography

**DOI:** 10.3390/molecules23092258

**Published:** 2018-09-05

**Authors:** Ninad Doctor, Yu Yang

**Affiliations:** Department of Chemistry, East Carolina University, Greenville, NC 27858, USA; doctorn11@students.ecu.edu

**Keywords:** aspirin, metformin HCl, high performance liquid chromatography, HPLC, subcritical water chromatography, SBWC

## Abstract

Organic solvents are widely used in pharmaceutical and chemical industry for chromatographic separations. In recent years, subcritical water chromatography (SBWC) has shown ability in replacing hazardous organic solvents used in traditional high-performance liquid chromatography (HPLC). In this work, a pain killer—aspirin—and an antidiabetic drug—metformin HCl—were successfully separated on an XBridge C18 column using no organic solvents in the subcritical water chromatography mobile phase. Both traditional HPLC and subcritical water chromatography were used for comparison purposes. SBWC separation of metformin HCl and aspirin were achieved at 95 °C and 125 °C, respectively. The recovery for both active pharmaceutical ingredients (APIs) obtained by SBWC is 99% in comparing with the stated content of each drug. The relative standard deviation is less than 1% for SBWC assays developed in this work. This level of accuracy and precision achieved by SBWC is the same as that resulted by the traditional HPLC analysis.

## 1. Introduction

Aspirin is a nonsteroidal anti-inflammatory drug [1,2,3]. *Prostaglandins* are produced in the cells by the enzyme cyclooxygenase (COX) and have several important functions. There are two COX—COX1 and COX2—in the human body and they both promote inflammation, pain, and fever. Nonsteroidal anti-inflammatory drugs (NSAIDs) block the COX enzymes and reduce prostaglandins throughout body, resulting in reducing ongoing inflammation, pain, and fever. Metformin HCl is widely used in treating type 2 diabetes [4,5,6]. It reduces hepatic (liver) glucose production, decreases GI glucose absorption, and increases target cell insulin sensitivity.

High performance liquid chromatography (HPLC) is predominantly used for analysis of both pharmaceutical active ingredients investigated in this study. HPLC methods rely heavily on organic solvents that are not only costly, but the storage and disposal of the HPLC wastes after the analysis are also very expensive [7,8,9].

As we all know, water at room temperature is very polar. The solubility of moderately polar and nonpolar organics in ordinary water is too low. Thus, pure ambient water cannot be used as the extraction fluid or chromatography mobile phase component for separation of most organic compounds. Fortunately, the polarity of water can be lowered by heating. The dielectric constant of water is decreased from 79 to below 30 when the temperature is increased from 25 °C to 250 °C [10,11,12]. Please note that the dielectric constant of methanol and acetonitrile is higher than 30. This means that subcritical water at 250 °C has lower polarity than pure methanol or acetonitrile. Similarly, the surface tension and viscosity of water are also significantly decreased when water is heated. Therefore, water at 200–250 °C should behave like a polar organic solvent. Because of the lowered polarity of high-temperature water, the solubility of nonpolar organic compounds in water can be enhanced to tens of thousands- to hundreds of thousand-fold by simply increasing the temperature from ambient to 200–250 °C [10,11]. The decreased polarity, surface tension, and viscosity makes it possible to use subcritical water as the sole mobile phase component and achieving reversed phase liquid chromatographic separation. This new technique is called subcritical water chromatography (SBWC) [10,11,12]. SBWC uses only water or buffer in the mobile phase and has a great potential to eliminate or reduce the usage of toxic and expensive organic solvents in separation technique [10,11,12,13,14,15,16,17,18,19,20,21,22,23,24,25]. In recent years, researchers have shown industrial applications of isothermal and thermal gradient separation of active pharmaceutical ingredients (APIs) using subcritical water chromatography [26,27,28,29,30,31,32]. In our group, several SBWC methods have been developed for analysis of various APIs in cold drugs and active ingredients in skincare products. The analytes investigated include dextromethorphan hydrobromide, chlorpheniramine maleate, acetaminophen and phenylephrine hydrochloride, niacinamide, preservatives, and sunscreens [33].

In pharmaceutical industry, the requirement for research and development department is to develop and validate analytical methods to quantify the APIs in drug dosage before the drug leaves the plant. While these standard HPLC methods work well for pharmaceutical companies, toxic organic solvents are required. Subcritical water chromatography method development can be capable of abolishing the use of hazardous organic solvents from the mobile phase for certain APIs, and then reducing the cost of analysis and waste disposal. Therefore, SBWC is classified as a green chromatographic technique.

In this work, green subcritical water chromatography methods were developed for analysis of aspirin and metformin HCl. Different temperatures and mobile phase combinations have been studied to optimize the separation conditions. Drug tablets were used to investigate the accuracy and precision of API quantifications. The results of SBWC analyses were also compared with those obtained by traditional HPLC to further evaluate the quality of SBWC separation and analysis.

## 2. Experimental

### 2.1. Reagents and Materials

Phosphoric acid was purchased from EMD Millipore Corporation (Billerica, MA, USA). Acetonitrile, formic acid (90%), sodium chloride, and phenol were obtained from Thermo Fisher Scientific (Fair Lawn, NJ, USA). Acetylsalicylic acid was received from ACROS Organics (Fair Lawn, NJ, USA). Ammonium hydroxide solution was purchased from Spectrum Chemical (New Brunswick, NJ, USA). Sodium 1-heptanesulfonate was received from Sigma-Aldrich (St. Louis, MO, USA).

Glass vials were acquired from Supelco (Bellfonte, PA, USA). Enteric coated aspirin pain killer was purchased from local pharmacy. Metformin HCl standard and metformin HCl extended release tablets were received from Amneal Pharma (New York, NY, USA). XBridge column (C18, 3.5 µm, 4.6 × 100 mm) was obtained from Waters Inc. (Milford, MA, USA).

### 2.2. Preparation of Internal Standard and Working Standard Solutions

For aspirin analysis, phenol served as an internal standard. The internal standard solution was prepared by adding 0.1000 g of phenol into a 100-mL volumetric flask, then dissolved and diluted to the mark with diluent. The diluent solution was prepared using acetonitrile:formic acid (99:1). A working standard solution was prepared by transferring 0.0500 g of acetylsalicylic acid into a 100-mL volumetric flask. Then 5 mL of internal standard was added into the volumetric flask and diluted to the mark with diluent.

For metformin HCl separation and analysis, phenol was again used as an internal standard. Metformin HCl stock solution was prepared by transferring 0.0500 g of metformin HCl into a 100-mL volumetric flask. It was dissolved and diluted to the mark with a different diluent that was prepared by dissolving 1.25% acetonitrile into 1 L water. Then 10 mL of the stock solution and 5 mL of internal standard were transferred into another a 100-mL flask and the solution was diluted to the mark with the same diluent.

### 2.3. Preparation of Sample Solutions

Each aspirin tablet content is 81 mg as found on the label. Ten tablets of aspirin were weighed on a balance to find out how much tablet powder would require to be equivalent to 50 mg of aspirin. All tablets were crushed into fine powder with the help of mortar and pestle. 0.0630 g of the fine powder was transferred into a 100-mL volumetric flask. The powder was dissolved and diluted to the mark with diluent. 10 mL of the aspirin sample was transferred into another 100-mL volumetric flask. Then, 5 mL of internal standard was added and diluent was used to fill the flask to the mark before HPLC analysis.

Each metformin HCl tablet contains 1000 mg metformin HCl as found on the label. In order to find out how much tablet powder would be required to produce 50 mg of metformin HCl, five tablets of metformin HCl were weighed. All tablets were crushed into fine powder using mortar and pestle. 0.0550 g of that fine powder was transferred into a 100-mL of volumetric flask, and then it was dissolved and diluted to the mark with diluent. 10 mL of the metformin HCl sample solution was transferred into another 100-mL volumetric flask. Then, 5 mL of internal standard was added and diluent was used to fill the flask to the mark before HPLC analysis.

### 2.4. Subcritical Water Chromatography and Traditional HPLC

Shimadzu Nexera UFLC with a UV–vis dual wavelength detection system (Shimadzu Corporation, Tokyo, Japan) was used for the separation and analysis of aspirin and metformin HCl. Aspirin was detected at 254 nm and metformin HCl was detected at 218 nm. The same HPLC system was used to carry out both traditional HPLC and the newly developed SBWC experiments.

For HPLC analysis of aspirin, acetonitrile was set at 15% in line B and line A was phosphoric acid buffer with pH 3.4. For SBWC separations temperature was set to 125 °C. The flow rate was 1.5 mL/min for both HPLC and SBWC separations.

In HPLC analysis of metformin, HCl acetonitrile was set in line B at 13% and line A was 0.06 M phosphoric acid buffer with pH 3.85. For SBWC separations at 95 °C, 100% phosphoric acid buffer with pH 3.85 was employed as the sole mobile phase component. Therefore, hazardous organic solvents are completely eliminated, and thus, subcritical water chromatography is a truly green separation technique. The flow rate was 1.0 mL/min for both HPLC and SBWC separations.

## 3. Results and Discussion

### 3.1. Separation and Analysis of Aspirin

**Figure 1:**
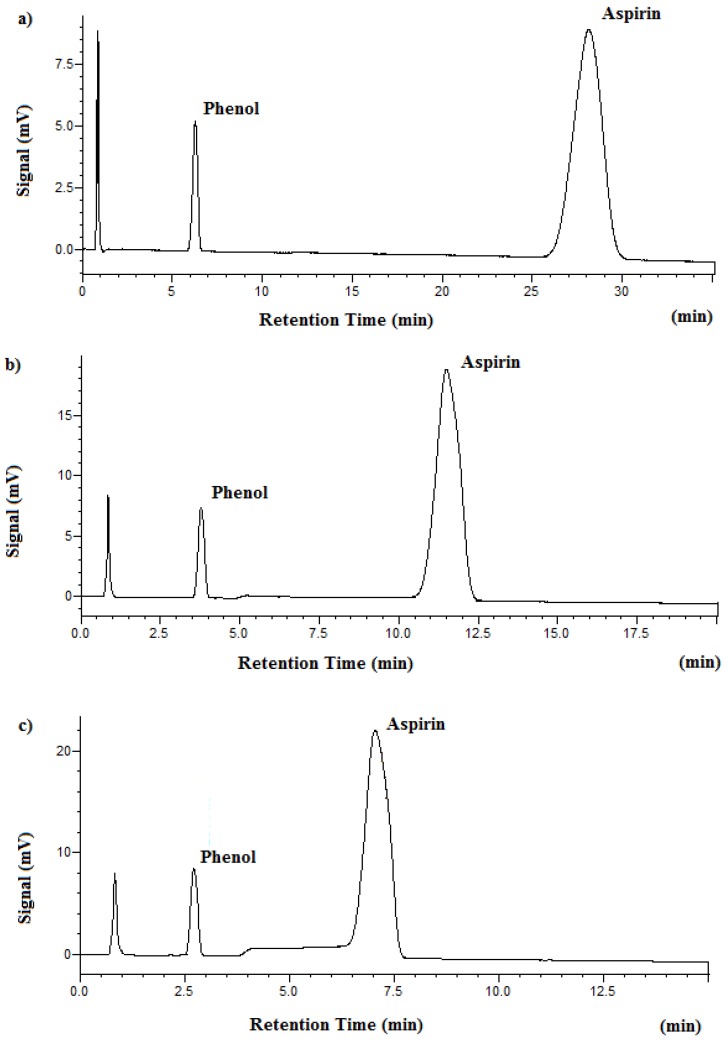
SBWC chromatograms of aspirin obtained on the XBridge C18 column with 1.5 mL/min and UV detection at 254 nm. Mobile phase was 100% phosphate buffer at pH 3.4. (**a**) 95 °C, (**b**) 125 °C, (**c**) 150 °C.

In order to achieve green separation and analysis of aspirin by subcritical water chromatography, various buffer solutions and temperatures have been evaluated. Figure 1 shows the chromatograms of aspirin at three different temperatures while Table 1 summarizes the temperature effect on SBWC pressure, retention time, and plate number. As one can easily see, the system pressure is lowered with increasing temperature. This is due to the decreased viscosity of water at higher temperatures as discussed earlier in the Introduction section. The decreased viscosity leads to lowered system pressure when the temperature rises. The retention time is shortened when the separation temperature is raised. The reason for this is that the polarity of water is greatly decreased with increasing temperature, making subcritical water more compatible with aspirin, and thus creating faster elution at higher temperatures. The peak width is also decreased with increasing temperature because the viscosity is decreased at higher temperatures. The lower viscosity allows faster mass transfer that results in narrower peaks. Our results show that the phosphate buffer at pH 3.4 and the separation temperature at 125 °C can achieve good separation using subcritical water chromatography. Phenol served as an internal standard owing to its thermal stability at high temperatures. Traditional HPLC separations at room temperature were conducted using 85% phosphate buffer at pH 3.4 and 15% acetonitrile in the mobile phase. The results of HPLC analysis of aspirin are needed to further evaluate the quality of SBWC separation and analysis of aspirin.

**Figure 2:**
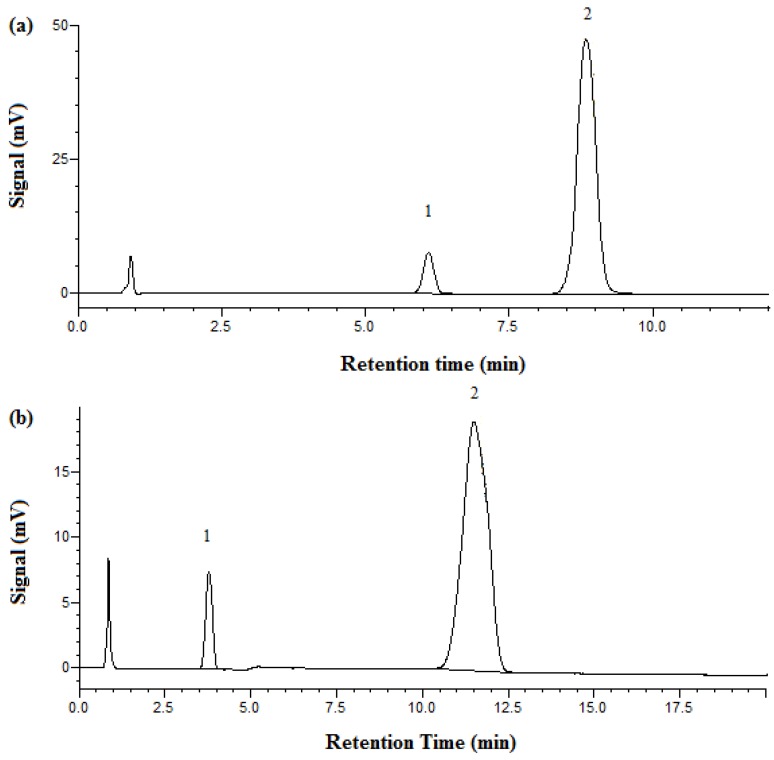
Chromatograms of aspirin obtained on the XBridge C18 column with 1.5 mL/min and UV detection at 254 nm. (**a**) HPLC at 25 °C, mobile phase: 85% phosphate buffer at pH 3.4 and 15% acetonitrile. (**b**) SBWC at 125 °C, mobile phase: 100% phosphate buffer at pH 3.4. Peak identification: 1. phenol, 2. aspirin.

Figure 2 shows the chromatograms of aspirin obtained by both HPLC and SBWC. At room temperature with HPLC, phenol retention time was observed at 6.1 min and aspirin at 8.8 min. However, under SBWC conditions at 125 °C and with 100% phosphate buffer (no acetonitrile) in the mobile phase, phenol was eluted early at 3.4 min and aspirin was eluted at 11.5 min.

Experiments were also conducted to evaluate the analytical merits of this newly developed subcritical water chromatography method for aspirin analysis and the following was found. The detection limit (LOD) is 1 µg/mL, or 1 ppm. The lower limit of quantification (LOQ) is 5 µg/mL, or 5 ppm. The upper limit of quantification is 50,000 µg/mL, or 50,000 ppm. Therefore, the linear range is 4 orders of magnitude. The regression coefficient is 1, while the slope is 1731 (y = 1731x + 31,460).

The accuracy of the separation and analysis of aspirin was evaluated using percent recovery. It was calculated by dividing the quantity of aspirin found using HPLC or SBWC by the quantity of aspirin used for analysis according to the stated content on the label of the drug, and then times 100.

As shown in Table 2, excellent aspirin recovery was achieved by both high-performance liquid chromatography and subcritical water chromatography. The percent RSDs were less than 1 for both traditional HPLC and SBWC analyses. The quantification results demonstrated that our SBWC analysis of aspirin are accurate, precise, and compare very favorably with the results reported in [7]. In that work, the accuracy of aspirin HPLC analysis was 97–103% and the precision in CV (%RSD) was 1–4% [7]. The good accuracy and precision of our results also mean that there was no aspirin degraded during the short SBWC run. It must be pointed out that the quality of SBWC separation and analysis is as good as that of the traditional HPLC as shown in Table 2. 

### 3.2. Separation and Analysis of Metformin HCl

Like the SBWC separation of aspirin, the pressure is reduced, retention time is shortened, and peaks becomes narrower with increasing temperature. For separation and analysis of metformin HCl by subcritical water chromatography, 100% phosphate buffer at pH 3.85 and separation temperature at 95 °C can achieve good separation. Phenol was used again as the internal standard for metformin HCl. In order to evaluate the quality of SBWC separation and analysis of metformin HCl, HPLC separations at room temperature were carried out using 87% phosphate buffer at pH 3.85 and 13% acetonitrile in the mobile phase.

**Figure 3:**
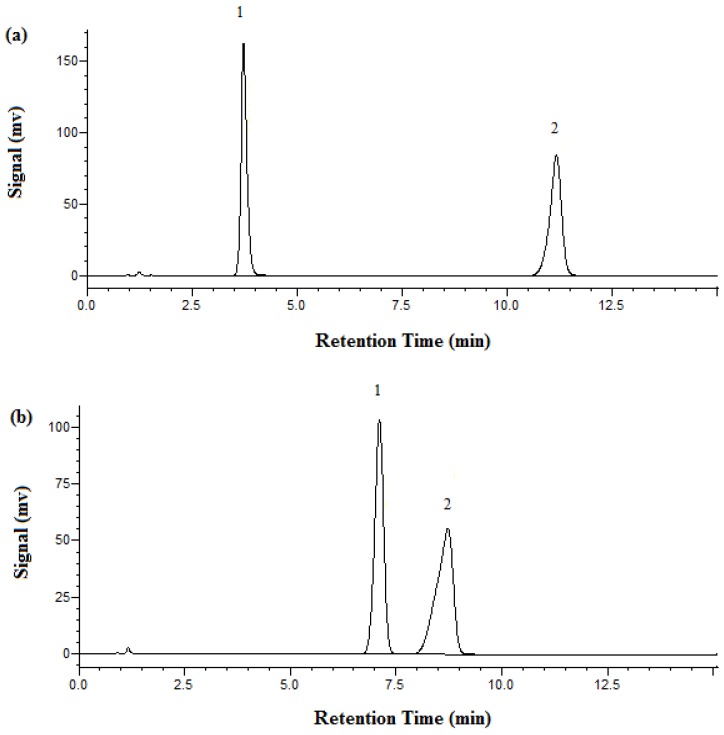
Chromatograms of metformin HCl obtained on the XBridge C18 column with 1 mL/min and UV detection at 218 nm. (**a**) HPLC at 25 °C, mobile phase: 87% phosphate buffer at pH 3.85 and 13% acetonitrile. (**b**) SBWC at 95 °C, mobile phase: 100% phosphate buffer at pH 3.85. Peak identification: 1. metformin, 2. phenol.

Figure 3 shows the chromatograms of metformin obtained by both HPLC and SBWC. At room temperature with HPLC, metformin HCl’s retention time was 3.7 min and phenol’s 11.2 min. A retention time for metformin of 7.2 min was achieved under SBWC conditions at 95 °C and with 100% phosphate buffer as the mobile phase, while phenol was eluted at 8.7 min. 

We also carried out experiments to evaluate the analytical merits of this newly developed subcritical water chromatography method and found out the following: the detection limit (LOD) is 0.005 µg/mL, or 5 ppb. The lower limit of quantification (LOQ) is 0.05 µg/mL, or 50 ppb. The upper limit of quantification is 5000 µg/mL, or 5000 ppm. Thus, the linear range is 5 orders of magnitude. The slope of the linear regression line is 29,272 (y = 29,272x + 614,386), while the correlation coefficient is 0.9992.

The quantification results for metformin HCl are given in Table 3. Nearly 100% metformin HCl recovery was achieved by both high-performance liquid chromatography and subcritical water chromatography. The percent RSDs were very low, less than 1 for both traditional HPLC and SBWC analyses. Our SBWC quantification quality is at least as good as that achieved by other researchers using traditional HPLC methods [8]. For example, an accuracy of 98–106% and precision (%RSD) of 2–8% were reported in [8]. Again, the quality of SBWC separation and analysis compare very favorably with that of the traditional HPLC as shown in Table 3. This also demonstrates no metaformin degradation during the short SBWC run.

## 4. Conclusions

Separation of aspirin and metformin HCl has been achieved on an XBridge C18 column using subcritical water chromatography at 125 °C and 95 °C, respectively. Since 100% phosphate buffer was employed as the only mobile phase component in the SBWC methods developed in this study, toxic and expensive acetonitrile that is required by traditional HPLC methods is eliminated. The percent recovery of both aspirin and metformin HCl achieved by SBWC is 99% and the RSDs are less than 1%, demonstrating that this green separation technique for separation and analysis of aspirin and metformin HCl is accurate and precise. The quality level of the quantification obtained by SBWC is as good as that achieved by traditional HPLC.

## Figures and Tables

**Table 1:** Temperature effect on SBWC pressure, retention time, and plate number.

	Temperature (°C)	Retention Time (min)	Plate Number
Aspirin	95 °C	28.2	1368
Phenol	95 °C	6.28	2278
Metformin	95 °C	7.11	5086
Aspirin	125 °C	11.5	1021
Phenol	125 °C	3.78	1580
Aspirin	150 °C	7.05	655
Phenol	150 °C	2.73	1099

**Table 2:** Comparison of aspirin quantification results achieved by traditional HPLC and SBWC.

Method	Amount of API Used (mg)	Amount of API Found (mg)	%Recovery	%RSD ^a^
Traditional HPLC	63.7	63.1	99.0	0.7
SBWC (125 °C)	63.5	63.1	99.3	0.6

^a^ Based on three replicate measurements.

**Table 3:** Comparison of metformin HCl quantification results achieved by traditional HPLC and SBWC.

Method	Amount of API Used (mg)	Amount of API Found (mg)	%Recovery	%RSD ^a^
Traditional HPLC	57.4	57.1	99.6	0.3
SBWC (95 °C)	54.2	53.5	98.8	0.4

^a^ Based on three replicate measurements.
